# Enhanced Osteogenesis of Dental Pulp Stem Cells In Vitro Induced by Chitosan–PEG-Incorporated Calcium Phosphate Cement

**DOI:** 10.3390/polym13142252

**Published:** 2021-07-09

**Authors:** Jae Eun Kim, Sangbae Park, Woong-Sup Lee, Jinsub Han, Jae Woon Lim, Seung Jeong, Myung Chul Lee, Woo-Young Yang, Hoon Seonwoo, B. Moon Kim, Yun-Hoon Choung, Kyoung-Je Jang, Jong Hoon Chung

**Affiliations:** 1 Department of Biosystems Engineering, Seoul National University, Seoul 08826, Korea; je6740@snu.ac.kr (J.E.K.); rhineop@snu.ac.kr (J.H.); 2 Department of Biosystems & Biomaterials Science and Engineering, Seoul National University, Seoul 08826, Korea; sb92park@snu.ac.kr (S.P.); jwlim1130@snu.ac.kr (J.W.L.); jsw3055@snu.ac.kr (S.J.); 3 Department of Chemistry, College of Natural Sciences, Seoul National University, Seoul 08826, Korea; kabigon@snu.ac.kr (W.-S.L.); kimbm@snu.ac.kr (B.M.K.); 4 BK21 Global Smart Farm Educational Research Center, Seoul National University, Seoul 08826, Korea; 5 Department of Brigham and Women’s Hospital, Division of Engineering in Medicine, Harvard Medical School, Cambridge, MA 02139, USA; josephmyungchul@gmail.com; 6 Dental Research Institute, Seoul National University, Seoul 08826, Korea; yang0829@snu.ac.kr; 7 Department of Industrial Machinery Engineering, College of Life Sciences and Natural Resources, Sunchon National University, Suncheon 57922, Korea; uhun906@gmail.com; 8 Interdisciplinary Program in IT-Bio Convergence System, Sunchon National University, Suncheon 57922, Korea; 9 Department of Otolaryngology, Ajou University School of Medicine, Suwon 16499, Korea; yhc@ajou.ac.kr; 10 Division of Agro-System Engineering, College of Agriculture and Life Science, Gyeongsang National University, Jinju 52828, Korea; 11 Institute of Agriculture & Life Science, Gyeongsang National University, Jinju 52828, Korea; 12 Global Smart Farm Convergence Major, Seoul National University, Seoul 08826, Korea; 13 Research Institute of Agriculture and Life Sciences, Seoul National University, Seoul 08826, Korea

**Keywords:** calcium phosphate cement, chitosan-poly (ethylene glycol) (CS/PEG), dental pulp stem cell, osteogenesis, bone substitute

## Abstract

The use of bone graft materials is required for the treatment of bone defects damaged beyond the critical defect; therefore, injectable calcium phosphate cement (CPC) is actively used after surgery. The application of various polymers to improve injectability, mechanical strength, and biological function of injection-type CPC is encouraged. We previously developed a chitosan–PEG conjugate (CS/PEG) by a sulfur (VI) fluoride exchange reaction, and the resulting chitosan derivative showed high solubility at a neutral pH. We have demonstrated the CPC incorporated with a poly (ethylene glycol) (PEG)-grafted chitosan (CS/PEG) and developed CS/PEG CPC. The characterization of CS/PEG CPC was conducted using Fourier transform infrared spectroscopy (FT-IR) and X-ray diffraction (XRD). The initial properties of CS/PEG CPCs, such as the pH, porosity, mechanical strength, zeta potential, and in vitro biocompatibility using the WST-1 assay, were also investigated. Moreover, osteocompatibility of CS/PEG CPCs was carried out via Alizarin Red S staining, immunocytochemistry, and Western blot analysis. CS/PEG CPC has enhanced mechanical strength compared to CPC, and the cohesion test also demonstrated in vivo stability. Furthermore, we determined whether CS/PEG CPC is a suitable candidate for promoting the osteogenic ability of Dental Pulp Stem Cells (DPSC). The elution of CS/PEG CPC entraps more calcium ion than CPC, as confirmed through the zeta potential test. Accordingly, the ion trapping effect of CS/PEG is considered to have played a role in promoting osteogenic differentiation of DPSCs. The results strongly suggested that CS/PEG could be used as suitable additives for improving osteogenic induction of bone substitute materials.

## 1. Introduction

Musculoskeletal disorders, such as osteoporosis, are chronic conditions that indicate the deterioration of bone tissue and loss of strength. Each year, over two million bone grafting surgical procedures are performed worldwide; thus, osteoporotic fractures are major clinical challenges [1]. Bone grafting is used to repair and regenerate bones through the transplantation of bone tissue materials [2]. Bone defect treatment is generally performed using autologous and allogeneic bone grafting. However, clinical results for the complete regeneration of bone tissue are not readily available. Limited donor supply and the morbidity of donor sites are typical drawbacks of autologous bone grafting. In addition, allogeneic bone grafting transplantation carries a risk of immune rejection and potential disease transmission, resulting in serious harm to the patient’s health.

Calcium phosphate cement (CPC), which was developed in 1986 by Brown and Chow, is used as an alternative method to other types of bone grafting due to its advanced bioactive properties, such as biocompatibility, moldability, and bone conductivity [3,4]. CPC has many applications in orthopedic and periodontic operations [5]. CPC enables noninvasive therapy as it can be injected by syringe into the affected area to fill in the damaged bone defects and stabilize the site [6]. A common characteristic of CPC, which is a hydraulic cement, is its conversion into a dough-like moldable compound that is held in a solid mass after mixing the calcium phosphate powder with an aqueous phase, which involves a dissolution-precipitation reaction [7,8]. The final product of CPC is distinguished depending on the setting reaction: hydroxyapatite or brushite CPC [8]. The final CPC compounds, such as hydroxyapatite (HA-CPC), have higher biological affinity and mechanical strength than brushite CPC. However, despite improvements in the clinical application of CPC, low mechanical strength resulted in severe clinical safety issues [9]. From the perspective of CPC, tricalcium phosphate (TCP, Ca_3_(PO_4_)_2_) and HA are well-known for having bone-binding properties, but the reason why these calcium phosphate-based ceramics exhibit physiological activity in living bone tissue has not yet been clarified. In addition, the behavior of the bone defects varies depending on the properties of the ceramics; hence, their composition and crystallinity are actively studied [10].

Hybrid systems involving the incorporation of various materials into CPC, such as bioactive molecules, hydrogels, polymers, and bioactive glass, are likely to yield favorable bone regeneration outcomes. Among these materials, chitosan is a promising candidate for using bone regeneration materials with CPC [11,12]. It is a versatile natural polymer that is a deacetylated derivative of chitin, which is one of the most abundant naturally occurring polysaccharides [13,14]. Chitosan exhibits biodegradability, biocompatibility, and antimicrobial activity, and has accelerated wound-healing properties with osteogenesis for bone regeneration [15,16]. Moreover, chitosan is able to chemically functionalize by combining various compounds [17,18]. Xu et al. [12] reported that chitosan-incorporated CPC improves mechanical properties and expands its application range. However, chitosan is poorly soluble, except in acidic media; thus, the incorporation of an acidic chitosan solution in CPC results in an increase in the pH, which can induce an inflammatory response [19,20]. 

We previously developed a chitosan derivative, soluble at a neutral pH, which was synthesized from coupling chitosan–ESF (CS/ESF) and mPEG–NH_2_ to form a chitosan–PEG conjugate (CS/PEG), grafting chitosan with PEG–NH_2_ by a sulfur (VI) fluoride exchange (SuFEx) reaction, as reported by Sharpless et al. [21]. The development of modified chitosan with PEG materials is based on the pegylation process. In addition, a stable sulfonamide linkage prepared by SuFEx click reaction was incorporated. This bond is known as a biostere of amide bond used for peptides and proteins, and is expected to have higher stability in vivo because it is a more stable and stronger bond than amide [22]. This biomaterial exhibited sustained release properties and has been reported in an application study on carrier properties with drug delivery potential [23]. For the internal 3D structure of the CS/PEG-derived supramolecular hydrogels, it was characterized that they had stacked-like network structures, which indicates their good capability to entrap ions as well as carry drugs.

The present study further investigated the application of CS/PEG in CPC to enhance the osteogenic differentiation of human dental pulp stem cells (DPSCs). CPC is widely used in dental applications, and chitosan-based materials have also been extensively studied [17,24]. Stem cells are an important resource for various medical applications in tissue regeneration engineering [18]. DPSCs are mesodermal-derived dental stem cells that are capable of self-renewal and can differentiate into various types of cells [16,25]. In particular, the possibility of osteogenic differentiation of DPSCs has been documented [26]. The aim of the present study was to develop a modified CPC incorporating CS/PEG (CS/PEG CPC) as an additive to improve the osteoconductivity and bioactivity of bone substitute materials. We evaluated the effect of CS/PEG on the mechanical and biological properties of CPC. The hypotheses were as follows: (1) CS/PEG would increase the mechanical strength of the CS/PEG composite compared with CPC, which influences its handling properties, moldability, and biocompatibility, (2) DPSCs would attach to CS/PEG CPC with improved cell proliferation and migration rates, and (3) CS/PEG CPC would support osteogenic differentiation under the DPSC microenvironment. The CS/PEG CPC could be considered as an alternative biomaterial with advantageous properties, including bioactivity and osteoconductivity, thus acting as an effective therapeutic agent for osteoporosis. Therefore, the influence of CS/PEG on CPC was investigated with a focus on osteogenesis differentiation in this study.

## 2. Materials and Methods

### 2.1. Fabrication of CS/PEG CPC

The preparation procedures for the synthesis of chitosan–ESF (CS/ESF), mPEG–NH_2_, and chitosan–mPEG (CS/PEG) were described in our previous study [23]. Low molecular weight chitosan (molecular weight 50,000–190,000 Da) were procured from Sigma-Aldrich (St. Louis, MO, USA). The CS/PEG CPC was prepared from a mixture of α-tricalcium phosphate (α-TCP; Ca_3_(PO_4_)_2_), dicalcium phosphate anhydrous (DCPA; CaHPO_4_), calcium carbonate (CaCO_3_), nanocrystalline hydroxyapatite (nHA; Ca_10_(PO_4_)_6_(OH)_2_), and 4% disodium phosphate (Na_2_HPO_4_) solutions. CS/PEG CPC liquids were prepared with chitosan/PEG solution mass fractions of 0, 1, 2.5, 5, 7.5, and 10 wt.%. The composition of CS/PEG CPC and the formulation of the CS/PEG solution are presented in Table 1 and Table 2, respectively. CPC powders (g) and different concentrations of CS/PEG solution (mL) were mixed at room temperature for 2 min and then placed in different sized molds. All samples were incubated for three days in a humidified CO_2_ incubator at 37 °C.

### 2.2. Characterization of CS/PEG CPC

#### 2.2.1. Anti-Washout Ability

An anti-washout test was used to determine the water resistance of CS/PEG CPC. Each prepared sample was placed in a 35 mm polystyrene dish filled with 5 mL of a sterilized phosphate-buffered saline solution (PBS; Welgene Inc., Gyeongsan, Korea) and kept in a humidified CO_2_ incubator at 37 °C for 24 h. The samples were then visually evaluated [27].

Mass loss(%) = (W_0_ − W_1_)/W_0_ (1)

#### 2.2.2. pH Measurement

To create experimental conditions similar to the physiological environment, the pH of CS/PEG CPC was evaluated. All samples were soaked in PBS (0.01 mol/L, pH = 7.4, Welgene Inc., Gyeongsan, Korea) and kept in an incubator at 37 °C for 28 days, after which the pH was measured using a pH meter (FE20, Mettler Toledo, Greifensee, Switzerland). The PBS was changed every three days.

#### 2.2.3. Scanning Electronic Microscopy (SEM)

The surface morphologies of CS/PEG CPC were observed using field-emission scanning electron microscopy (FE-SEM; SUPRA 55VP, Carl Zeiss, Oberkochen, Germany). The CS/PEG CPCs were sputter-coated with platinum and observed at an accelerating voltage of 2 kV.

#### 2.2.4. Fourier Transform Infrared (FT-IR)

Fourier transform infrared (FT-IR) spectra of CS/PEG CPC were measured in the range from 1600 to 400 cm^−1^. FT-IR spectra were recorded on a PerkinElmer Spectrum Two spectrophotometer (Waltham, MA, USA) for solid and liquid samples. 

#### 2.2.5. Determination of Porosity

The porosity was measured as previously described in the literature [28,29]. The sample (Φ 6 × 2 mm^2^) was placed in a pycnometer filled with ethylene glycol and then placed under a vacuum for 1 h to remove the bubbles. The total porosity was calculated using Equation (2).

(2) porosity(%)=100*(A4−A1)/(A2+A4−A3)

where A1 is the initial weight of the CS/PEG CPC, A2 is the weight of the pycnometer filled with ethylene glycol, A3 is the weight of the pycnometer with the CS/PEG CPC, and A4 is the weight of the CS/PEG CPC after discarding the ethylene glycol in the pycnometer.

#### 2.2.6. Adsorption of Bovine Serum Albumin (BSA)

To evaluate the BSA adsorption capacity of the different concentrations of CS/PEG on CPC, 1% BSA was dissolved in 1 mL of PBS (0.01 M, pH = 7.4) at 37 °C. The prepared 5% CS/PEG CPC samples and 0% CS/PEG CPC samples (Φ 6 × 2 mm^2^) as a control group were placed in a 96-well plate soaked in BSA solution, and then placed in an incubator for 24 h. After removing the solution, the attached protein was evaluated in 50 μL of BSA using a BCA protein assay kit (Sigma-Aldrich, St. Louis, MO, USA). All tests were repeated five times [30].

#### 2.2.7. Compressive Strength

The compressive strength was used to determine the mechanical properties of the CPC. A sample with 8 mm in diameter and 10 mm in height was prepared. A universal testing machine (TA. HDi; Stable Micro Systems, UK) was used at a test speed of 0.2 mm/s to measure the compressive strength. The maximum load required to fracture each sample was measured. The total compressive strength was calculated using Equation (3) [31].

(3) $$\sigma_{b}\left(MPa\right)=\frac{p_{b}}{A_{b}}$$

where $\sigma_{b}$ is bearing stress, and $A_{b}$ indicates the characteristic area perpendicular to compressive load ($p_{b})$.

#### 2.2.8. X-ray Diffractometry (XRD)

The crystalline phases formed during the CS/PEG CPC setting reaction were analyzed using an X-ray diffractometer (D8 Advance, Bruker, Breme, Germany) with Cu-Kα radiation at 3 kW. The intensities of the peaks at 30.8° (2θ) for α-TCP and 25.9° (2θ) for HA were recorded.

#### 2.2.9. Zeta Potential

Zeta potential measurements were performed using a Zetasizer 3000 (Malvern Panalytical, Herrenberg, Germany). PBS was used as the liquid phase. The potential was determined to be three times the mean value, and the standard deviation was calculated.

### 2.3. In Vitro Experiments

Human Dental Pulp Stem Cells (DPSCs) were provided from the Tooth Bioengineering National Laboratory at the College of Dentistry, Seoul National University (IRB: CRI05004). The DPSCs were seeded onto the CPC with different concentration ratios of CS/PEG (*w/v*, 0% and 5%). Cells were incubated with alpha-modified Eagle’s medium (α-MEM; Welgene Inc., Gyeongsan, Korea) supplemented with 10% fetal bovine serum (FBS; Welgene Inc., Gyeongsan, Korea), 2 mM L-glutamine, 100 U/mL penicillin, and 100 μg/mL streptomycin (Gibco BRL, Carlsbad, CA, USA) at 37 °C in a humidified atmosphere containing 5% CO_2_. For the osteogenic differentiation of DPSCs, the cells were substituted with osteogenic differentiation conditioned media, which consisted of α-MEM supplemented with 10% FBS, 1% penicillin, 0.1 µM dexamethasone (Sigma-Aldrich, St. Louis, MO, USA), 10 mM β-glycerophosphate (Sigma-Aldrich, St. Louis, MO, USA), and 100 µM ascorbic acid (Sigma-Aldrich, St. Louis, MO, USA). The culture medium was changed every three days. 

#### 2.3.1. Cytotoxicity Assay

Different concentration w/v ratios (0% and 5%) of CS/PEG CPC samples were sterilized in an autoclave at 120 °C for 1 h and placed in a 96-well plate. The DPSCs were incubated in a 6-well plate at a density of 3 × 10^4^ cells/well. After reaching 70–80% confluence, the cells were sub-cultured and placed on the CS/PEG CPC in the wells. The cytotoxicity was evaluated using a water-soluble tetrazolium salt assay kit (WST-1; Dogenbio, Seoul, Korea). After incubation for one and seven days, the cells were washed twice with PBS and incubated in medium with 10% WST-1 reagent for 1 h. The absorbance was measured using a microplate reader at a wavelength of 450 nm. Three samples from each group were measured, and the data are displayed as the mean ± standard deviation.

#### 2.3.2. Alizarin Red S Staining

The calcium deposition of the DPSCs seeded with CS/PEG CPC was evaluated using Alizarin Red S (ARS) staining. First, the DPSCs were seeded on a 6-well plate (5 × 10^4^ cells/well) with different concentration ratios of CS/PEG CPC samples (0% and 5%) and incubated for 24 h in a humidified CO_2_ incubator. Subsequently, the DPSCs were substituted with osteogenic differentiation conditioned media. On days 7 and 14 of the differentiation, the cells were fixed with 4% paraformaldehyde for 30 min. The fixed cells were treated with a 2% ARS solution for 30 min at room temperature, followed by washing with deionized water. Images of the stained cells were acquired using a microscope (Nikon, Tokyo, Japan). The stained cells were de-stained with a 10% cetylpyridinium chloride/10 mM sodium phosphate solution and then incubated at room temperature for 30 min. The absorbance of the de-stained solution was measured using a microplate reader at a wavelength of 570 nm.

#### 2.3.3. Immunocytochemistry

To confirm the expression of the osteogenic markers of the DPSCs on the CS/PEG CPC, the DPSCs were seeded on a 96-well plate (2 × 10^3^ cells/well) with different concentration ratios of CS/PEG CPC samples (0% and 5%) and incubated for 24 h in a humidified CO_2_ incubator. Subsequently, the DPSCs were substituted with osteogenic differentiation conditioned media on days 7 and 14. Afterward, the cells on the CS/PEG CPC were fixed with a 4% paraformaldehyde solution (Sigma-Aldrich, St. Louis, MO, USA) for 30 min at room temperature. The samples were treated with 0.2% Triton X-100 (Sigma-Aldrich, St. Louis, MO, USA) for 15 min, and then stained with TRITC-conjugated phalloidin (Millipore, Burlington, MA, USA) for 1 h and 4’,6-diamidino-2-phenylindole (DAPI;Millipore, Burlington, MA, USA ) for 5 min. The osteopontin (OPN) protein was stained with monoclonal anti-OPN antibody (1:500, Abcam, Cambridge, MA, USA) and FITC-conjugated goat anti-human antibody for 1 h. A Nikon fluorescence microscope was used to acquire images of the stained cells. Quantitation analysis of expression of OPN was carried out using ImageJ software (NIH, Bethesda, MD, USA).

#### 2.3.4. Western Blot

Western blotting was conducted to confirm the expression of runt-related transcription factor 2 (Runx2) in DPSCs cultured on CS/PEG CPC. DPSCs were seeded on the CS/PEG CPC at a concentration of 5 × 10^4^ cells/well. After three days, the culture medium was removed and replaced with osteogenic differentiation conditioned media. Proteins were harvested at one and two weeks of osteogenic differentiation. Briefly, the culture media was removed, and each sample was washed with prechilled PBS. The cells were subsequently harvested from the CS/PEG CPC and treated with a cell lysis buffer (Millipore, Burlington, MA, USA), followed by incubation and centrifugation. The supernatant was collected and separated by 8% sodium dodecyl sulfate-polyacrylamide gel electrophoresis (SDS-PAGE) under reducing conditions. The separated proteins were transferred to a polyvinylidene fluoride (PVDF) membrane (Millipore) at 30 V for 1 h. The expression level of Runx2 was observed using a ChemiDoc Imaging System (Bio-Rad Laboratories, Hercules, CA, USA). All data were quantitatively analyzed using ImageJ software (NIH, Bethesda, MD, USA).

### 2.4. Statistical Analysis

Statistical analyses were performed using R Studio for Windows v1.2.5042 (RStudio Inc., Boston, MA, USA). Statistical significance between the samples was compared using one-way ANOVA at * *p* < 0.05. The data were reported as the mean ± standard deviation (*n* = 5).

## 3. Results

### 3.1. Characterization at Different Amounts of CS/PEG in CPC

**Figure 1:**
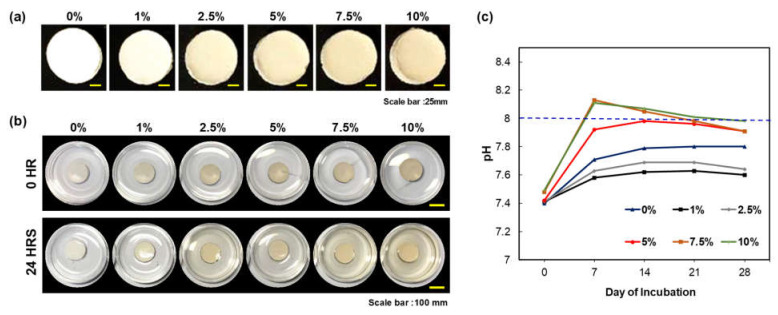
Characterization of CS/PEG CPC. (**a**) Image of CS/PEG on the CPC with five different concentrations of CS/PEG. All CS/PEG CPC samples were tested at 0 and 24 h of incubation in PBS. (**b**) Image of CS/PEG CPC samples. (**c**) Change in pH of CS/PEG CPC with time (incubated at 37 °C for 28 days; PBS was changed every three days).

The cohesion of the CS/PEG CPC was investigated by observing the washout resistance (Figure 1a). None of the samples were broken or washed out with PBS. Furthermore, as the concentration of CS/PEG in CPC increased, the release of chitosan increased (Figure 1a, b). The pH of the incubated PBS containing CS/PEG CPC was monitored for 28 days (Figure 1c). On day 7 of the incubation, the pH of the 7.5% and 10% CS/PEG CPC was higher than that in the other samples. After day 7, the pH gradually decreased with the incubation time and reached a near-neutral pH. However, the pH of the 7.5% and 10% CS/PEG exceeded 8. In the 5% CS/PEG CPC, the pH did not increase to 8, which would potentially have no physiological effect [32,33]. 

**Figure 2:**
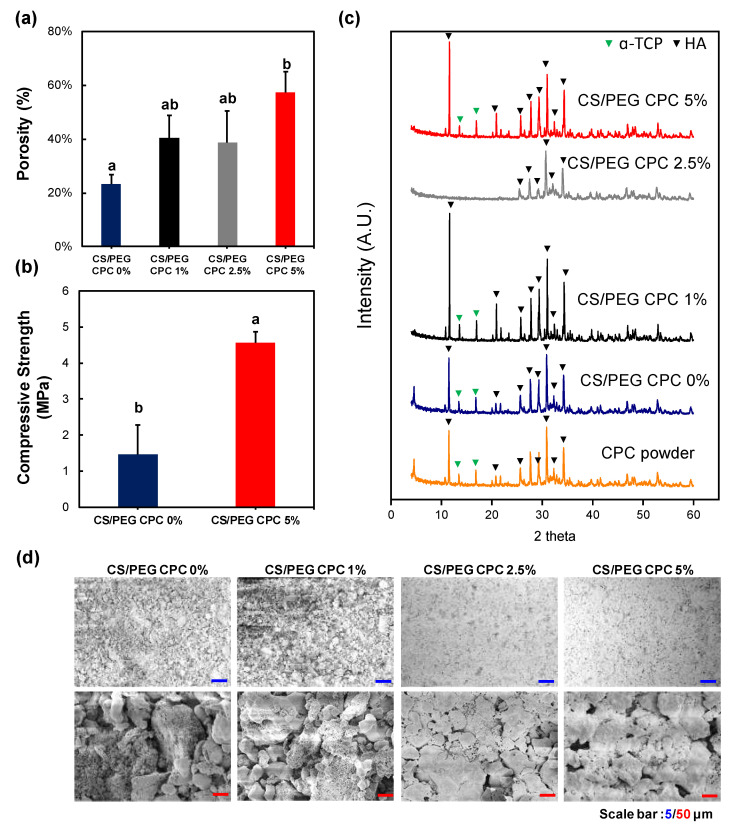
Physical properties of CS/PEG CPC. (**a**) Overall porosity of CS/PEG CPC. (**b**) The mechanical properties of the CS/PEG CPC were analyzed using a texture analyzer. (**c**) X-ray diffraction patterns of the CPC powder and CS/PEG CPC identify the crystalline phases in the material and thereby reveal the chemical composition. The positions of the referenced peaks of HA (JCPD No. 9-432) are indicated. (**d**) Scanning electron microscope (SEM) images of surface morphology of CS/PEG CPC that were hardened for 3 days (*n* = 5, ANOVA, Duncan’s multiple range test, *p* < 0.05). Same letters indicate that there is no significant difference between samples.

**Figure 3:**
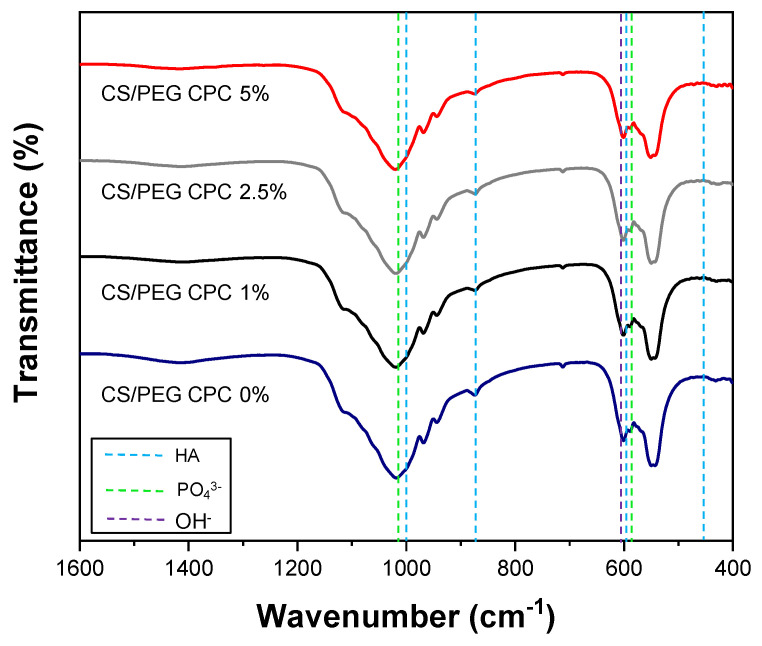
Fourier transform infrared (FT–IR) spectra of CS/PEG CPC was recorded for the hydroxyl group (625 cm^−1^) and the phosphate groups (1112, 1030, 960, 605, and 563 cm^−1^) [34,35].

The porosity of the CS/PEG CPC is presented in Figure 2a. The 0% CS/PEG CPC had the lowest porosity value compared to the other samples, and the 5% CS/PEG CPC had the highest value. Even though the porosity value of the 5% CS/PEG CPC was almost twice as high as the 0% CS/PEG CPC, it can be seen in Figure 2b that the compressive strength was significantly stronger than the 0% CS/PEG CPC (*p* < 0.05). The XRD patterns of the CPC before (CPC powder) and after (synthesized CS/PEG CPCs) are shown in Figure 2c. The CPC powder exhibits a low HA peak with a high α-TCP, whereas the CS/PEG CPC shows a higher HA; thus, the XRD analysis confirmed that the CPC powder was synthesized in both. Scanning electron microscope (SEM) images of surface morphology of CS/PEG CPC are shown in Figure 2d. As the concentration of CS/PEG solution increased, the microstructure of CS/PEG CPC agglomerated to form a layer on the surface of CS/PEG CPC. Chemical analysis of functional groups of specific bonds in the sample was performed using infrared spectroscopy (FT-IR). The FT-IR spectrum of the CS/PEG CPC samples confirms that all samples exist in an identical stretch. In Figure 3, the characteristic peaks exhibited the stretching mode of hydroxyl group (625 cm^−1^), and the phosphate groups (1112, 1030, 960, 605, and 563 cm^−1^) indicate the confirmation of the typical peaks characteristic of CPC [34,35]. In addition, CS/PEG CPCs were thermally stable when treated at high temperature (Figure S1). 

### 3.2. Biocompatibility of DPSCs on CS/PEG CPC

**Figure 4:**
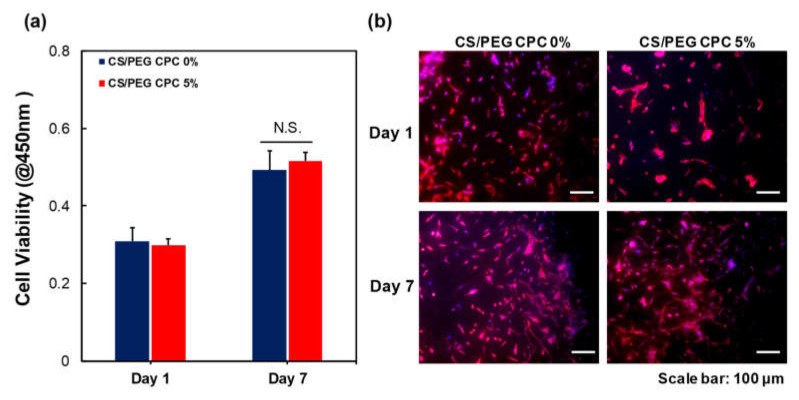
Biocompatibility of the 0% and 5% CS/PEG CPC. (**a**) Cell viability of DPSCs on days 1 and 7 of culture using a WST-1 assay (*n* = 5, ANOVA, Duncan’s multiple range test, *p* < 0.05). N.S. indicates no significant difference. (**b**) Immunocytochemistry staining images of DPSCs. F-actin was stained with TRITC phalloidin (red) and nuclei were stained with DAPI (blue).

To analyze the cell viability, the WST-1 assay was performed using the DPSCs. The cells were cultured on the 0% and 5% CS/PEG CPC for seven days and exhibited an increase in cell proliferation; however, there was no significant difference in the cell viability between the 0% and 5% CS/PEG CPC (Figure 4a). In Figure 4b, the morphologies of the DPSCs on the CPC by immunocytochemistry (ICC) after seven days of culture. F-actin (red) and DAPI (blue) indicate the cytoskeleton and nucleus, respectively. It can be seen that the DPSCs are well-attached to the CS/PEG CPC. The images also show that the CS/PEG CPC exhibits good cell adhesion and biocompatibility. 

### 3.3. Osteogenic Differentiation of DPSCs on CS/PEG CPC

**Figure 5:**
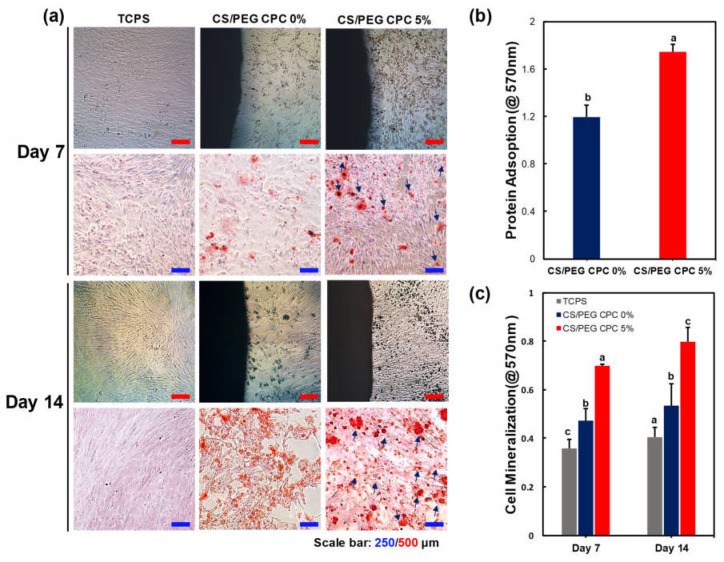
Mineralization by CS/PEG CPC surrounding microenvironment analyzed by ARS staining. DPSCs were cultured on the 0% and 5% CS/PEC CPC, with tissue-cultured polystyrene (TCPS) as the control. (**a**) Image of the cells cultured for 7 and 14 days in osteogenic differentiation media: the calcium nodules exhibit denser and darker (red) staining with increasing culture time. The calcium nodule formation is indicated (blue arrow). (**b**) BSA protein absorption of 0% and 5% CS/PEG CPC after incubation for one day (*n* = 5, ANOVA, Duncan’s multiple range test, *p* < 0.05). (**c**) Quantification of calcium nodules by ARS staining. Different letters indicate significant differences between groups (*n* = 5, ANOVA, Duncan’s multiple range test, *p* < 0.05).

As the CS/PEG concentration in the CPC increased, the CS/PEG dissolved and was released in the PBS, as shown in Figure 1a. Accordingly, an ARS experiment was conducted to confirm the effect of the released CS/PEG on the osteogenic differentiation of the DPSCs. Thus, the cells around the CS/PEG CPC were cultured in osteogenic media for 7 and 14 days. The cell morphology and ARS staining of the calcium nodules (red) are shown in Figure 5a, and the DPSCs surrounding the 5% CS/PEG CPC showed greater staining in the microscopic analysis. According to Figure 5c, the 5% CS/PEG CPC showed a significant effect on the formation of mineralized calcium nodules. Moreover, the BSA protein adsorption was examined to determine the bonding behavior. The 5% CS/PEG CPC samples were incubated on the BSA solution for one day, after which the BSA protein was significantly more adsorbed than 0% CS/PEG CPC (Figure 5b). The influence of the CS/PEG substrates on the zeta potential of the CPC is shown in Table 3. Zeta potential refers to the potential difference between the dispersion medium and the fixed fluid layer attached to the dispersed particles. The particle size, polydispersity index (particle size distribution), and zeta potential values were obtained for the CPC samples. The average zeta potentials of the 0% and 5% CS/PEG CPC were −9.53 ± 1.44 and −8.70 ± 0.33 mV, respectively. 

**Figure 6:**
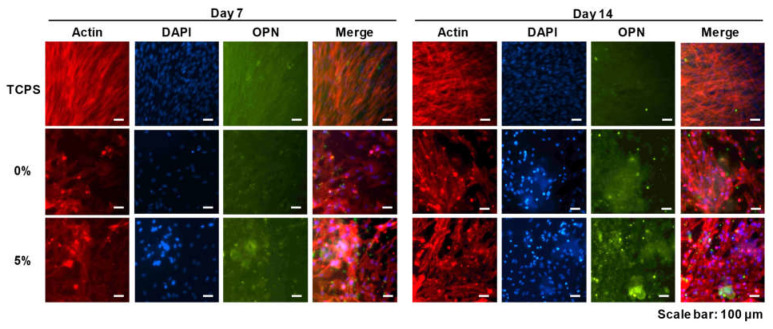
Immunofluorescence images of the CS/PEG CPC at 7 and 14 days. Actin, osteopontin (OPN), and cell nuclei (DAPI) were exhibited as red, green, and blue, respectively. Scale bars: 100 µm.

To further investigate the osteogenic differentiation of the DPSCs on CS/PEG, immunocytochemistry (ICC) was performed to observe the osteopontin (OPN), which can be upregulated during the osteogenic differentiation of cells (Figure 6). The quantification analysis of OPN expression is shown in Figure S2. DPSCs were cultured for 14 days under osteogenic media. The ICC images showed that the DPSCs in the culture dish (control) were attached and homogeneously distributed, but the expression of OPN was low compared to DPSCs cultured on the CS/PEG CPCs. In contrast, the cell morphology of the DPSCs on the CS/PEG CPC was not homogeneous and the osteogenic differentiation was enhanced, particularly in the 5% CS/PEG CPC. Moreover, fluorescent OPN (green) was highly expressed in the 5% CS/PEG CPC at day 14. 

**Figure 7:**
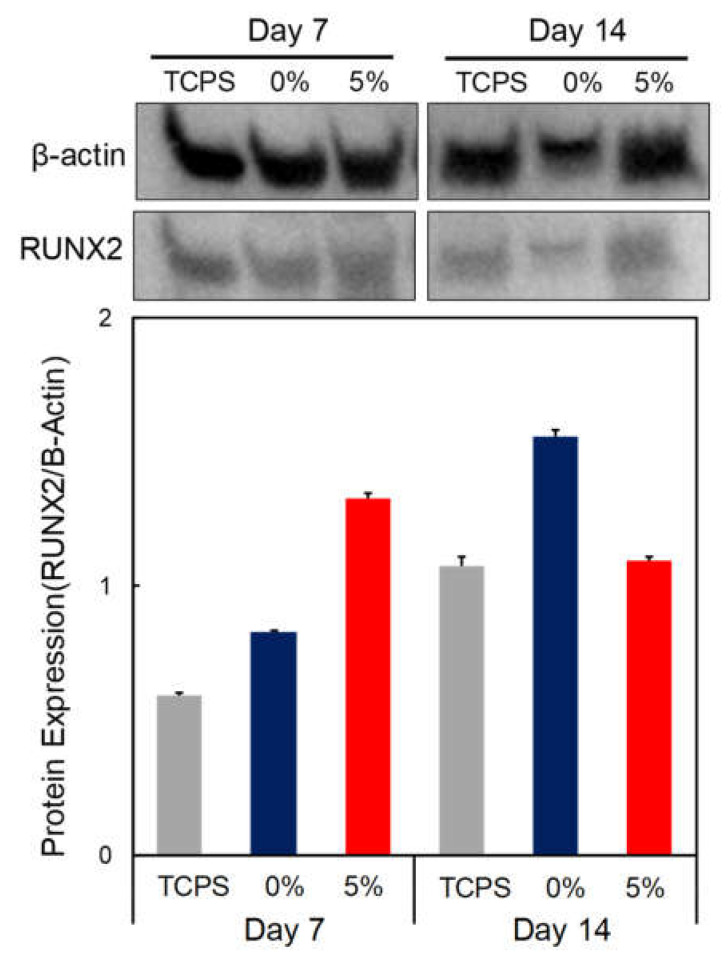
Western blot analysis of DPSCs seeded on CPC. Osteogenic gene expression, Runx2 expression, of CPC at days 7 and 14 of culture was analyzed by Western blot. Β-actin was employed as an inner control.

The Western blot analysis to investigate the effects of the osteogenic differentiation of DPSCs cultured on the 0% and 5% CS/PEG CPC is shown in Figure 7. A comparison of the CS/PEG CPC with the control group (TCPS) and the 5% CS/PEG CPC showed higher gene expression levels of the osteo-progenitor marker Runx2 at day 7, but the value decreased at day 14.

## 4. Discussion

Although CPC is a well-known bone grafting biomaterial, it still lacks sufficient biocompatibility and osteoconductivity. There are many processing challenges associated with the optimization of calcium phosphate cements. Our experimental results demonstrate the effects of incorporating CPC with CS/PEG and the role of CS/PEG in the synthesis of calcium phosphate cements. Modifying chitosan with PEG exhibits solubility under neutral conditions and can be utilized as an additive for the liquid component in CPC. The calcium phosphate ionizes and dissolves with a hardening accelerator (sodium phosphate dibasic, Na_2_HPO_4_) in the paste state [36]. After the ions are supersaturated, a dissolution-precipitation reaction occurs between the calcium phosphate particles that are bonded and solidified [37]. The hardening accelerator generates HPO_4_^2−^ ions, which are the same ions as those generated by α-TCP when ionized. Since the crystal growth in CPC depends on the dissolution-precipitation reaction, the hardening accelerator plays a critical role in precipitating the calcium and phosphate ions to form hydroxyapatite [8,38]. CS/PEG was developed to dissolve under neutral conditions by combining PEG through a click reaction to overcome the disadvantage of chitosan, which is soluble only under acidic conditions. Therefore, increasing the concentration of CS/PEG in the hardening accelerator is considered to enhance the interaction between CS/PEG and CPC due to potent Ca^2+^-chelating properties of PEGylated biomolecules. According to the literature, the solubility of calcium phosphate compounds is high in acidic and basic regions and low in neutral regions [26,39]. As shown in Figure 1c, by adding CS/PEG to CPC, the pH was higher than neutral. Thus, this phenomenon can be explained by the increase in the solubility of calcium phosphate, which means that the dissolution of α-TCP and the recrystallization to hydroxyapatite is likely to increase. In addition, Cheng et al. [40] demonstrated that a basic environment could promote the earlier nucleation of hydroxyapatite (HA) which was formed through the setting reaction of CPC. Thus, increasing the nucleation of HA is considered to support the mineralization of CS/PEG CPC. 

The micromorphology of CPCs was shown in SEM images, where CS/PEG CPC showed a more homogeneous surface with porous structure. According to Gbureck et al. [41], chitosan influenced cement, which dispersed more homogeneously due to their higher surface charge. These results are possibly related mechanical properties. Barralet et al. [31] also suggested a chelate reaction between citric acid (e.g., glucose, chitosan) and calcium, which might tightly interlock crystal and could increase in strength. The effect of chitosan on the FT-IR spectrum of the CS/PEF CPC confirms the existence of hydroxyapatite through the peak of the 630 cm^−1^ hydroxyl group (OH). Based on the zeta potential analysis, the average particle size presented a smaller distribution than that of the 0% CS/PEG CPC, and more dissolution occurred in the CS/PEG CPC. However, the 7.5% and 10% CS/PEG are not suitable for clinical application because the pH rises faster and exceeds 8 (Figure 1c). Due to the resulting pH rise with the increase of concentrations of CS/PEG, two possible explanations were presented. First, a chitosan monomer bears a tertiary amino group, hence, solvated chitosan/PEG acts as a weak base and the amino groups can be protonated under PBS buffer solution (pH 7.4) to slightly increase pH. Next, as the chelating effect lowers ionic bond strength between calcium cation and phosphate anion, the elution of phosphate, biphosphate, and carbonate anions from CPC increases, that is, the eluted phosphate ion can be protonated under PBS buffer solution (pH 7.4) to increase pH. Due to the buffer capacity of PBS, a small amount of those eluted anions increase pH below 8.0 with 5% CS/PEG in CPC. On the other hand, the 5% CS/PEG CPC approached the neutral pH of the PBS solution after sufficient time elapsed. It is possible that the pH decreases during the recrystallization from hydroxyapatite to achieve solubility equilibrium by a buffering action [42]. In addition, the absolute zeta potential value of the 5% CS/PEG CPC (8.70 ± 0.33 mV) was lower than that of the 0% CS/PEG CPC (9.53 ± 1.44 mV). Colloidal stability is an important factor in aggregation and depends on the zeta potential. Due to the resulting zeta potential value of 0% and 5% CS/PEG CPCs below 10 mV, we presumed that the repulsive forces of the colloid particles decrease and the colloidal stability also decreases [43,44]. In other words, poor colloidal stability of CPCs led to particle aggregation, thus it is expected to precipitate with calcium ions, which affects the ionic strength.

In conclusion, it is expected that the CS/PEG material activates the colloid particles by reducing the absolute value of the zeta potential of the CPC and increasing the aggregation efficiency. In vitro experiments were performed in which DPSCs were cultured on the CS/PEG CPC. The results of the cell viability test showed no significant difference between the 0% and 5% CS/PEG CPC. This suggest that CS/PEG CPC does not affect the cell viability of DPSCs compared to conventional CPCs. In addition, the CS/PEG CPC does not contain or produce toxic substances. In the ARS experiments, the DPSCs cultured around the CPC showed that incorporating CS/PEG in CPC not only affects the osteogenic differentiation, but also affects the cell adhesion. Chitosan is a positively charged molecule that can interact with the negative part of the cell membrane, which can lead to the opening of tight junction proteins and thereby explains the permeation enhancing property of this polysaccharide [45]. The elution of CS/PEG in CPC can support osteoinduction. The CS/PEG CPC itself can induce osteoconduction, and the type of bone ingrowth of the pores depends on the material aspect of the effect of the pore size on bone regeneration, which affects the progression towards osteogenesis [46]. In addition, Ferrand et al. [47] verified a study in which chitosan-based biocomposites were associated with BMP2, BMP-7, or TGF-β1, which is well-known for inducing osteogenesis. A higher porosity is related to osteogenesis because it allows high oxygenation. Chen et al. [28] reported that cell proliferation and differentiation were dependent on the porosity of the scaffolds, and a porosity of 60% showed the best cell proliferation, osteogenic differentiation, and bone ingrowth. Our results were also confirmed through ARS, where the osteogenic differentiation was significantly high in the 5% CS/PEG CPC, with a porosity of 60%. Based on the BSA protein adsorption experiments, it is believed that this contributes to higher bone-inducing protein adsorption as well as ion exchange with apatite formation by dissolution and reprecipitation [48,49]. These results were likely due to the larger surface area, which resulted in a higher ion exchange and bone-inducing factor adsorption [49]. Bones generally have periodicity, while bone resorption and formation maintain homeostasis. Bone remodeling is the process of replacing old bones with new bones after growth is completed, and homeostasis is maintained according to the balance of osteoblast and osteoclast activity. Runx2 is a pre-osteoblast marker required for the differentiation of pre-osteoblasts into mature osteoblasts. Accordingly, the expression of Runx2 on the 5% CS/PEG at week 1 was higher than that in the other experimental groups because CS/PEG promoted osteogenic differentiation and activated the osteoblasts [50]. We performed physiological and biological assays to confirm whether the CS/PEG CPC provides the proper bone grafting materials. In conclusion, we demonstrated that the modification of calcium phosphate biomaterials with CS/PEG effectively enhances osteogenic differentiation. Addition of CS/PEG in the CPC may indicate substantial potential benefits for cell attachment and differentiation, and improve the osteogenic capability. However, future work is necessary for clinical application to overcome the inferior osteogenic capacity of CS/PEG, and the osteogenic gene expression will be further examined to assess the full potential of the osteogenesis of CS/PEG CPC. In addition, the phase composition of CS/PEG CPC was not characterized in vivo as a function of implantation. Future research will focus on these issues.

## 5. Conclusions

A comparative study between CPC and reinforced CPC by incorporating CS/PEG revealed improved osteoconductivity with enhanced mechanical strength and biocompatibility. The in vitro study showed that the CS/PEG CPC had sufficient biocompatibility and osteoconductivity for application as an alternative biomaterial for enhancing bone regeneration. The CS/PEG polymer is believed to have the potential to induce osteogenesis and shows promise for future application.

## Figures and Tables

**Table 1:** Composition of CS/PEG CPC.

Initial CPC Composition	Sample	Solution
60% α-Tricalcium phosphate (α-TCP; Ca_3_(PO_4_)_2_) 26% Dicalcium phosphate anhydrous (DCPA; CaHPO_4_) 10% Calcium carbonate (CaCO_3_) 4% Hydroxyapatite (HA)	CS/PEG CPC 0%	4% Na_2_HPO_4_
	CS/PEG CPC 1%	4% Na_2_HPO_4_ + CS/PEG 1%
	CS/PEG CPC 2.5%	4% Na_2_HPO_4_ + CS/PEG 2.5%
	CS/PEG CPC 5%	4% Na_2_HPO_4_ + CS/PEG 5%
	CS/PEG CPC 7.5%	4% Na_2_HPO_4_ + CS/PEG 7.5%
	CS/PEG CPC 10%	4 % Na_2_HPO_4_ + CS/PEG 10%

**Table 2:** Formulation of CS/PEG solution.

Solution	Mix Ratio of CPC
4% Na_2_HPO_4_ + CS/PEG powder (1%/2.5%/5%/7.5%/10%) in distilled water	Solution/Powder (L/P) ratio: 0.4 mL/g 0.4:1 (*v:w*)

**Table 3:** Zeta potential of 0% and 5% CS/PEG CPC.

Sample	Z-Average Particle Size (µm)	Zeta Potential (mV)
CS/PEG CPC 0%	6.174 ± 2.43	−9.53 ± 1.44
CS/PEG CPC 5%	1.338 ± 0.743	−8.70 ± 0.33

## Data Availability

All the experimental data presented herein are available upon request from the corresponding author.

## Supplementary Materials

The following are available online at https://www.mdpi.com/article/10.3390/polym13142252/s1, Figure S1: (a) The FT-IR spectra of CS/PEG CPC before and (b) after autoclaving (120 °C at 1 h). Figure S2. Quantification analysis of OPN expression.
