# Supplementary material for: Enhanced Osteogenesis of Dental Pulp Stem Cells In Vitro Induced by Chitosan–PEG-Incorporated Calcium Phosphate Cement
Source: Polymers (Basel). 2021 Jul 9;13(14):2252. doi: 10.3390/polym13142252 (PMC8309336; doi:10.3390/polym13142252)
Supplement: Supplementary file 1 [file polymers-13-02252-s001.zip › polymers-1296944-supplementary.pdf]

Supporting information

Enhanced Osteogenesis of Dental Pulp Stem Cells in vitro In-duced by Chitosan–PEG Reinforced Calcium Phosphate Cement

Jae Eun Kim[1†], Sangbae Park [2†], Woong-Sup Lee [3†], Jinsub Han[1,4], Jae Woon Lim[2], Seung Jeong[2], Myung Chul Lee[5], Woo-Young Yang[6], Hoon Seonwoo[7,8], B. Moon Kim[3], Yun-Hoon Choung[9], Kyoung-Je Jang[10,11,*] and Jong Hoon Chung[1,4,12,13,*]

[1] Department of Biosystems Engineering, Seoul National University, Seoul 08826, Korea; je6740@snu.ac.kr (J.E.K.); rhineop@snu.ac.kr (J.H.)
[2] Department of Biosystems & Biomaterials Science and Engineering, Seoul National University, Seoul 08826, Korea; sb92park@snu.ac.kr (S.P.); jwlim1130@snu.ac.kr (J.W.L.); jsw3055@snu.ac.kr (S.J.)
[3] Department of Chemistry, College of Natural Sciences, Seoul National University, Seoul 08826, Korea; kabigon@snu.ac.kr (W.S.L.); kimbm@snu.ac.kr (B.M.K.)
[4] BK21 Global Smart Farm Educational Research Center, Seoul National University, Seoul 08826, Korea; rhineop@snu.ac.kr
[5] Division of Engineering in Medicine Brigham and Women's Hospital Department Harvard Medical School, Cambridge, MA 02139, USA; josephmyungchul@gmail.com
[6] Dental Research Institute, Seoul National University, Seoul 08826, Korea; yang0829@snu.ac.kr
[7] Department of Industrial Machinery Engineering, College of Life Sciences and Natural Resources, Sunchon National University, Suncheon 57922, Korea; uhun906@gmail.com
[8] Interdisciplinary Program in IT-Bio Convergence System, Sunchon National University, Suncheon 57922, Korea; uhun906@gmail.com
[9] Department of Otolaryngology, Ajou University School of Medicine, Suwon 16499, Korea; yhc@ajou.ac.kr
[10] Division of Agro-System Engineering, College of Agriculture and Life Science, Gyeongsang National University, Jinju 52828, Korea;
[11] Institute of Agriculture & Life Science, Gyeongsang National University, Jinju 52828, Korea;
[12] Global Smart Farm Convergence Major, Seoul National University, Seoul 08826, Korea;
[13] Research Institute of Agriculture and Life Sciences, Seoul National University, Seoul 08826, Korea;

* Correspondence: jchung@snu.ac.kr (J.H.C.); kj_jang@gnu.ac.kr (K.-J.J.); Tel.: +82-2-880-4601 (J.H.C.); Tel.: +82-55-772-1898 (K.-J.J.)
† These authors contributed equally to this study.

Table of Contents

1.0 Thermal analysis of CS/PEG CPC at high temperature autoclave

2.0 Quantification analysis of expression of OPN

1.0 thermal analysis of CS/PEG CPC at high temperature autoclave

To analyze the thermal effect on CS/PEG CPC at high temperature, FT-IR spectra of CS/PEG CPC with various CS/PEG concentrations were recorded.

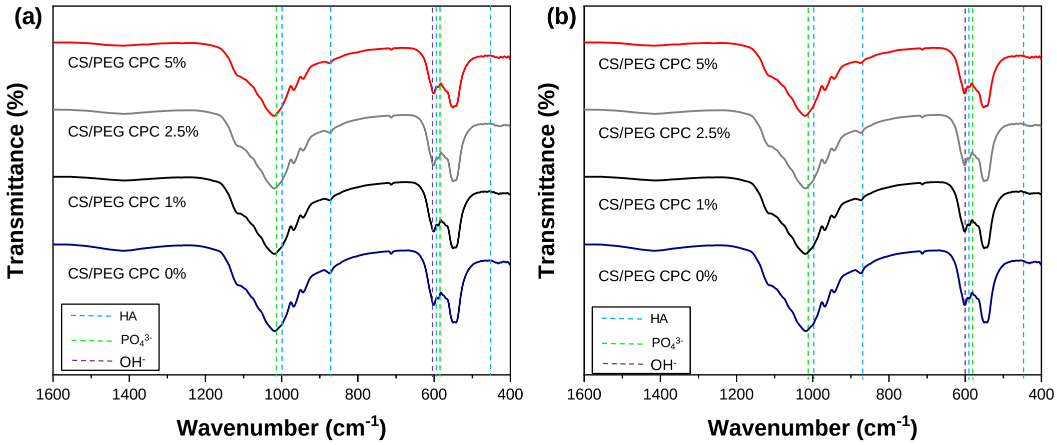

Figure S1. (a) The FT-IR spectra of CS/PEG CPC before and (b) after autoclaving (120°C at 1hours).

2.0 Quantification Analysis of OPN Expression

Quantification analysis of osteopontin expression (related to Figure 6). Comparing intensities of OPN with different samples using ImageJ. (n = 5, ANOVA, Duncan's multiple range test, $p < 0.05$). Same letters indicate that there is no significant. difference between samples.

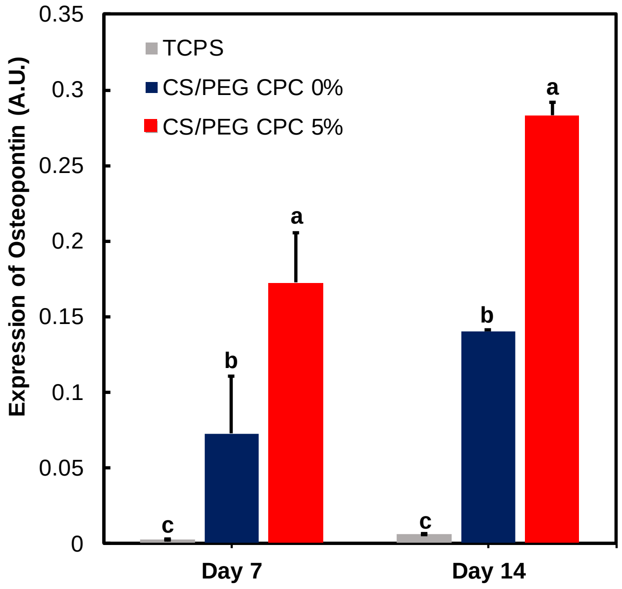

Figure S2. Quantification analysis of OPN expression
